# A Redox-Mediator-Integrated Flexible Micro-Supercapacitor with Improved Energy Storage Capability and Suppressed Self-Discharge Rate

**DOI:** 10.3390/nano11113027

**Published:** 2021-11-11

**Authors:** Sung Min Wi, Jihong Kim, Suok Lee, Yu-Rim Choi, Sung Hoon Kim, Jong Bae Park, Younghyun Cho, Wook Ahn, A-Rang Jang, John Hong, Young-Woo Lee

**Affiliations:** 1 Department of Energy Systems Engineering, Soonchunhyang University, Asan-si 31538, Korea; dnlals77@naver.com (S.M.W.); colorg11@naver.com (J.K.); solee0117@gmail.com (S.L.); uj1008@sch.ac.kr (Y.-R.C.); nyk4123@naver.com (S.H.K.); yhcho@sch.ac.kr (Y.C.); wahn21@sch.ac.kr (W.A.); 2 Jeonju Centre, Korea Basic Science Institute, Jeonju 54907, Korea; jbpjb@kbsi.re.kr; 3 Department of Electrical Engineering, Semyung University, Jecheon-si 27136, Korea; arjang@semyung.ac.kr; 4 School of Materials Science and Engineering, Kookmin University, Seoul 02707, Korea

**Keywords:** hydroquinone-based polymer-gel electrolyte, micro-supercapacitors, Faradaic redox reactions, energy storage

## Abstract

To effectively improve the energy density and reduce the self-discharging rate of micro-supercapacitors, an advanced strategy is required. In this study, we developed a hydroquinone (HQ)-based polymer-gel electrolyte (HQ-gel) for micro-supercapacitors. The introduced HQ redox mediators (HQ-RMs) in the gel electrolyte composites underwent additional Faradaic redox reactions and synergistically increased the overall energy density of the micro-supercapacitors. Moreover, the HQ-RMs in the gel electrolyte weakened the self-discharging behavior by providing a strong binding attachment of charged ions on the porous graphitized carbon electrodes after the redox reactions. The micro-supercapacitors with HQ gel (HQ-MSCs) showed excellent energy storage performance, including a high energy volumetric capacitance of 255 mF cm^−3^ at a current of 1 µA, which is 2.7 times higher than the micro-supercapacitors based on bare-gel electrolyte composites without HQ-RMs (b-MSCs). The HQ-MSCs showed comparatively low self-discharging behavior with an open circuit potential drop of 37% compared to the b-MSCs with an open circuit potential drop of 60% after 2000 s. The assembled HQ-MSCs exhibited high mechanical flexibility over the applied external tensile and compressive strains. Additionally, the HQ-MSCs show the adequate circuit compatibility within series and parallel connections and the good cycling performance of capacitance retention of 95% after 3000 cycles.

## 1. Introduction

Recent studies have demonstrated the potential of flexible micro-supercapacitors for supplying energy and electricity to future flexible and wearable electronics such as rollable displays, human-implanted devices, and high-end robotics [1,2,3]. The micro-supercapacitors are highly significant as future energy storage devices because they can be integrated with small-sized applications, operate under fast charge/discharge conditions, and have a long lifetime [4]. Moreover, developing an effective method to fabricate electrode structures on flexible substrates and depositing electrode materials on small areas is crucial for the successful utilization of micro-supercapacitors. As a result, tremendous efforts have been directed to develop carbon-based micro-supercapacitor electrode materials [5].

Carbon materials can be easily handled on flexible substrates, and their electrical and chemical properties are well tailored by a simple post-treatment process, inducing high electrochemical energy storage performance [6,7]. For example, the gold and nitrogen doping on the carbon electrode sample can increase the conductivity and wettability of the carbon electrode, inducing the improved electrochemical performance [8]. Moreover, Peng et al. reported that the boron doped laser-induced graphene has highly improved electrochemical performance, greater than the pure laser-induced graphene [9]. However, carbon-based micro-supercapacitors inevitably have a lower energy density than other energy storage systems because of their electrostatic/physical-only charge-storing kinetics [10,11]. In general, the energy density of carbon-based electrodes based on electric double layer capacitor (EDLC) lies in the range of 0.1~3 Wh kg^−1^ [12], but in a range of over 100 Wh kg^−1^ for Li ion batteries. There is also another type of supercapacitor (pseudocapacitors, with an energy density of about 10 Wh kg^−1^), but they store charges through Faradaic redox reactions on the surface of electrodes [13]. Moreover, carbon materials for flexible micro-supercapacitors based on EDLC suffer from a high self-discharging rate owing to the weak attachment of electrolyte ions on the carbon electrodes. Additionally, the polymer-gel electrolyte is another essential component to develop flexible and wearable micro-supercapacitors [14,15]. In general, the classic liquid-type electrolyte has critical issues to apply the flexible and wearable supercapacitors due not only to their electrolyte leakage but also to their high manufacturing costs, such as difficult packaging to fabricate flexible supercapacitors [16,17]. However, the pure polymer-gel electrolyte has the low ionic conductivity of the polymer medium [18,19]. Therefore, enhancing the energy storage performance and minimizing the self-discharging behavior are critical issues that must be resolved for carbon-based flexible micro-supercapacitors.

The prevalent carbon materials used in micro-supercapacitors are graphite-based 2D planar materials because of their outstanding electrical conductivity, highly tunable surface area, chemical stability, and mechanical behavior [20,21]. Therefore, many scientists have studied tailoring the three-dimensional morphology and surface functionalization of graphite materials to enhance their electrochemical properties [22,23]. Another strategy being investigated is the use of redox mediators (RMs) in gel electrolytes [24,25,26]. Especially, RMs can show high flexibility and mechanical/chemical stability when they are mixed with a gel electrolyte, as well as provide easy diffusion in the gel electrolyte. The addition of RMs plays pivotal roles in enhancing the performance of supercapacitors due to the induced electrochemical Faradaic redox reactions on the surface of electrodes, which can store more electron charges compared to double-layer capacitance [27,28]. Thus, the total capacitance of supercapacitors with redox mediators can store energy by both electric double layer capacitance and the pseudocapacitance working in parallel. Additionally, interestingly, RMs play key roles in minimizing the self-discharging behavior. In particular, Faradaic redox reactions of RMs result in a high binding attachment level of charged ions on carbon-based electrodes, and RMs increase the ionic conductivity of the gel electrolyte, inducing a low self-discharging rate. Therefore, introducing gel electrolyte composites with proper redox mediators might be crucial to further maximize the performance of carbon-based micro-supercapacitors. Especially, among various RMs, hydroquinone compounds can be regarded as one of the most promising redox-active mediators due to its small size and high electrochemical reversibility.

In this study, inspired by the highly interactive hydroquinone redox mediators (HQ-RMs), we systematically engineered composite mixtures with hydroquinone (HQ) as a redox mediator, polyvinyl alcohol (PVA) as a polymer-gel medium, and phosphoric acid as an acidic electrolyte (HQ-gel). The interdigitated graphite electrodes were fabricated by carbonization of polyimide (PI) sheets using a laser scribing method. The laser scribing method can be operated with a simple step process on polymer films (fast processing time) with good reproducibility by the systematic control of laser beams. Additionally, continuous fabrication on the polymer sheets is available. Finally, the carbon electrode materials can be simply deposited on the interdigitated structure by the induced carbonization from the polymer films. The assembled micro-supercapacitors with HQ-gel (HQ-MSCs) exhibit superior electrochemical performance, including a high volumetric capacitance of 255 mF cm^−3^, low self-discharge rate of an open circuit potential drop of 37% after 2000 s, and over 95% capacitance retention over 3000 charge/discharge cycles compared to the MSCs without the HQ-RMs (a volumetric capacitance of 94 mF cm^−3^_,_ self-discharging rate of an open circuit potential drop of 50% after 2000 s, and 90% capacitance retention over 3000 charge/discharge cycles). This enhancement might be attributed to the Faradaic redox reactions by the HQ-RMs and the strengthened adsorption of charged electrolyte ions on the carbon-based electrode. These findings demonstrate that the novel HQ-based gel electrolyte composites can be used to guarantee flexible carbon-based micro-supercapacitors with promising electrochemical energy storage performance for future wearable energy storage applications.

## 2. Materials and Methods

### 2.1. Fabrications of HQ-MSCs

For the HQ-MSCs, interdigitated carbon-based electrodes were directly fabricated by carbonization on PI films using a laser scribing method. The interdigitated carbon-based electrodes have seven fingers, and each electrode serves as both a working electrode and a current collector. This system does not require any separator because the interdigitated carbon-based electrodes are already separated on the PI film substrate with a length of 0.5 mm. For the electrolyte coating method, we prepared HQ-based polymer–gel electrolyte composites consisting of HQ (0.6 g, Sigma-Aldrich, Saint Louis, MO, USA) as a redox mediator, poly(vinyl alcohol) (PVA, Mw: 89,000–98,000, Sigma-Aldrich, Saint Louis, MO, USA), phosphoric acid (H_3_PO_4_, Sigma-Aldrich, Saint Louis, MO, USA), and deionized water (20 mL). The prepared HQ-based polymer–gel electrolyte was coated onto the interdigitated carbon-based electrodes and then dried overnight for stabilization.

### 2.2. Characterization and Electrochemical Tests of HQ-MSCs

We carried out powder XRD (Miniflex 600, Rigaku), Raman spectroscopy (iXR raman in Nexsa XPS system, Thermo Scientific, Korea Basic Science Institute-Jeonju Center), XPS (Nexsa XPS system, Thermo Scientific, Korea Basic Science Institute-Jeonju Center), and field-emission scanning electron microscopy (FE-SEM, Gemini SEM 300, ZEISS, Jena, Germany) analyses. In addition, the BET surface area of the samples was measured using nitrogen adsorption/desorption measurements (Belsorp mini X, MicrotracBEL Corp., Osaka, Japan). To confirm the deposition of the HQ-RMs, we performed Fourier transform infrared spectroscopy (FT-IR, TENSOR27, Bruker, NCIRF, Seoul National University-National Center for Inter-University Research Facilities, Billerica, MA, USA) analysis. The electrochemical capacitive behavior of the as-prepared MSCs was estimated using a potentiostat (PGSTAT302N, Metrohm, Autolab). The specific capacitance of the carbon electrodes was calculated by the GCD discharge curves. The specific areal capacitance was calculated by the discharge time and current density (mA/unit area), and the calculated specific areal capacitance was divided by the electrode thickness to evaluate the specific volumetric capacitance of the samples.

## 3. Results and Discussion

**Figure 1:**
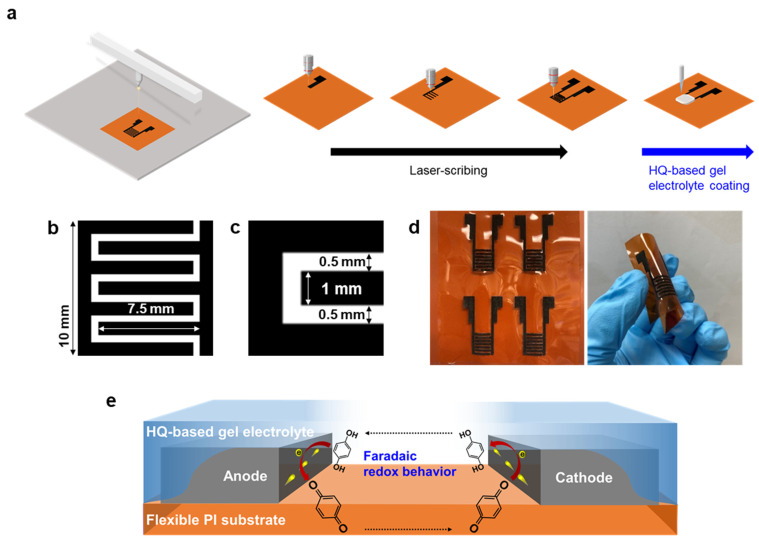
(**a**) Illustration of fabrication process for the interdigitated carbon-based micro-supercapacitors with HQ-gel (HQ-MSCs) based on a laser scribing method and HQ-based gel electrolyte. (**b**,**c**) Schematic image for pattern structure of MSCs. (**d**) Photograph of MSCs prepared by a laser scribing process. (**e**) Schematic illustration of electrochemical Faradaic redox behaviors of HQ in HQ-MSCs.

As shown in Figure 1a, the interdigitated carbon-based electrodes were fabricated using a stepwise direct laser scribing method on polyimide (PI) sheets. With direct laser irradiation, carbonization of the PI sheets immediately occurs using a pulsed laser and forms carbon-based electrodes. The interdigitated carbon-based electrodes were scribed on the PI sheets. After the laser-carbonization process, the HQ-gel composites were drop-coated onto the interdigitated carbon-based electrodes. Finally, the interdigitated carbon-based micro-supercapacitors with HQ-gel (HQ-MSCs) were dried overnight to stabilize the gel electrolyte. The interdigitated electrode structure used in the micro-supercapacitors is shown schematically in Figure 1b,c. Each finger has been designed by the fixed two-dimensional interdigitated structure (length of 7.5 mm and finger width of 1 mm). The gap distance between neighboring finger electrodes is ~0.5 mm. According to the cross-sectional SEM images (Figure S1), the electrodes show a thickness of 12 µm and the electrolyte layers have a thickness of 14 µm. Figure 1d shows the optical images of the fabricated HQ-MSCs on the PI sheets. Owing to the high flexibility of the PI sheets, the HQ-MSCs can sustain their original interdigitated MSC structure even when the PI substrates are strongly subjected to external bending forces. A cross-sectional schematic of the HQ-MSCs is shown in Figure 1e. On the PI substrates, two unconnected carbon-based electrodes were assigned to the symmetric anode and cathode electrodes. Both electrodes were covered by the HQ-gel composites. During the charge and discharge processes, the existence of the HQ-RMs in the polymer–gel electrolyte induces additional Faradaic redox reactions and delivers a high energy density compared to the micro-supercapacitors with the bare-gel electrolyte.

**Figure 2:**
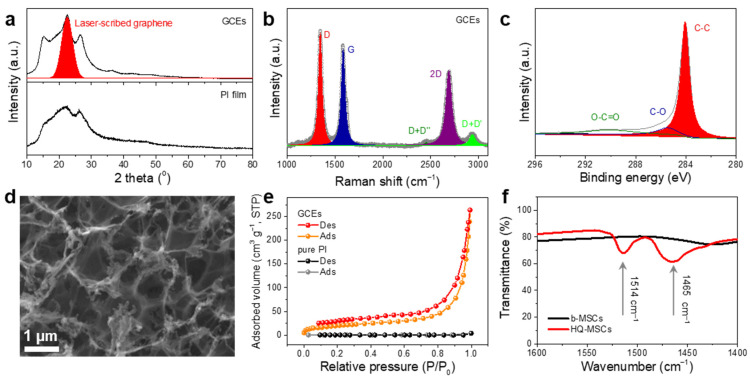
(**a**) X-ray diffraction (XRD) patterns of graphite-like carbon-based electrodes (GCEs) and pure polyimide (PI) film. (**b**) Raman spectrum and (**c**) X-rya photoelectron spectroscopy (XPS) C1s spectrum of GCEs. (**d**) Scanning electron microscopy (SEM) image of GCEs. (**e**) Nitrogen adsorption/desorption isotherm curves of GCEs and pure PI film. (**f**) Fourier transform infrared spectroscopy (FT-IR) spectra of HQ-MSCs and b-MSCs.

To evaluate the carbonization process by the crystallographic phase, X-ray diffraction (XRD) spectra of the pure PI films and carbon-based electrodes from the PI films were analyzed (Figure 2a). The clear XRD peaks at 15° and 27°, as well as broad intensity areas near 22.5°, were well matched with the crystal phase of PI [29]. After laser irradiation, new peaks at 23° were ascribed to the graphite-like carbon crystals after the carbonization process. The XRD spectrum of the graphite-like carbon-based electrodes (GCEs) exhibited a slight negative shift compared to that of the intrinsic graphite index. The peak shift can be attributed to the expanded d-spacing value of the GCEs resulting from the partial formation of the oxygen-containing functional group on the graphite layers during the laser carbonization process [30,31]. Figure 2b shows the Raman spectrum of the GCEs with three strong peaks at 1346 cm^−1^ (D-band), 1584 cm^−1^ (G-band), and 2689 cm^−1^ (2D-band) [32,33]. In general, the high ratio of I_2D_/I_G_ indicates the typical features of graphene. As the number of graphene layers increases, the ratio of I_2D_/I_G_ decreases. Thus, the I_2D_/I_G_ of graphite is commonly lower than that of graphene, which is less than 1 [34,35,36]. In this work, the I_2D_/I_G_ of GCEs is approximately 0.746, which demonstrates that the PI films were converted to graphite composites with two-dimensional layered structures. The existence of strong 2D-band peaks demonstrates that the PI films were converted to graphite composites with two-dimensional layered structures.

We also carried out X-ray photoelectron spectroscopy (XPS) measurements to determine the surface properties of the GCEs (Figure 2c). The GCEs exhibited a common graphite characteristic with a strong C-C peak clearly observed at 284.6 eV, which indicates a high degree of formation of layered graphite structures [37,38,39]. In addition, as shown in Figure 2d, the as-prepared GCEs showed a highly porous structure based on a three-dimensional network, owing to the rapid formation of gaseous species produced during laser irradiation. To further evaluate the specific surface area of the GCEs, Brunauer–Emmett–Teller (BET) measurements were carried out. As shown in Figure 2e, the GCEs exhibited typical adsorption/desorption curves of type II [40], and the calculated BET surface areas of the GCEs were observed to be 74.15 m^2^ g^−1^, which is higher than that of pure PI (0.08 m^2^ g^−1^). After the laser patterning process, the PI substrates were successfully converted to layered GCEs with large surface sites and good electrical conductivity, which are favorable for electrochemical energy storage. The measured electrical resistance of the GCEs by using the 2-probe method is approximately 55.6 Ω, whereas the resistance of the pure PI films was not measured due to the insulating properties (Figure S2).

In addition, the existence of the HQ-RMs in the polymer-gel electrolyte was investigated by Fourier transform infrared spectroscopy (FT-IR) spectra (Figure 2f). The HQ-MSCs showed two dominant peaks in the FT-IR spectrum, which corresponded to the phenyl ring stretching (1512 cm^−1^) and -C-OH in-plane bending (1465 cm^−1^) of HQ compared to MSCs without HQ-RMs (b-MSCs) [41]. These two clear peaks are the main signs that the HQ-RMs are well mixed in the gel electrolyte composites and are deposited on the GCEs. Thus, it is expected that the HQ-MSCs will exhibit improved energy-storing properties owing to their unique structural/electrochemical features as follows: (1) the well-designed graphite-like carbon-based interdigitated electrodes with good electrical conductivity, which supports the fast electron pathway; (2) the large surface area by porous structures, which provide large electrolyte contact areas; and (3) the induction of additional Faradaic redox reactions using HQ-RMs, which induce improved energy storage properties, as shown in Figure 1e. There is synergistic electrochemical contribution on the surface of the carbon electrodes (both electrical double-layer capacitance and Faradaic redox reactions by the HQ-RMs) [42,43,44].

**Figure 3:**
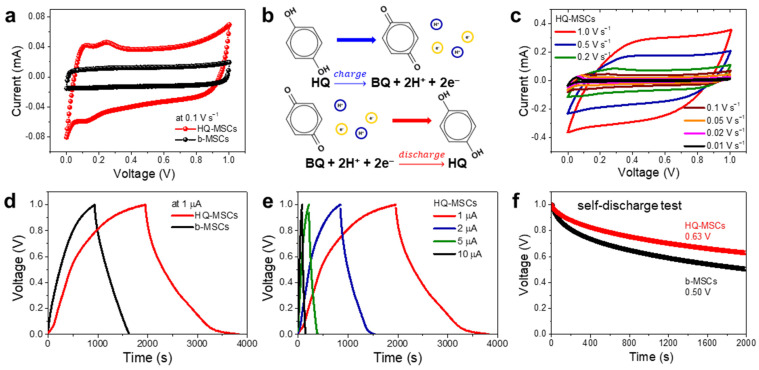
(**a**) Cyclic voltammetry (CV) curves of HQ-MSCs and b-MSCs at a scan rate of 0.1 V s^−1^. (**b**) Electrochemical Faradaic redox behavior mechanism between HQ and BQ. (**c**) CV curves of HQ-MSCs at different scan rates from 0.01 to 1.0 V s^−1^. (**d**) Galvanic charge/discharge graph (GCD) curves of HQ-MSCs and b-MSCs at a current 1 µA. (**e**) GCD curves of HQ-MSCs at different current ranges from 1 to 10 µA. (**f**) Self-discharging test of HQ-MSCs and b-MSCs after fully charged state.

The electrochemical properties of the HQ-MSCs were evaluated using a two-electrode system. Figure 3a shows the cyclic voltammetry (CV) curves of the HQ-MSCs and b-MSCs at a scan rate of 100 mV s^−1^. The area surrounded by the CV curve of the HQ-MSCs was larger than that of the B-MSCs, demonstrating a higher energy storage performance of the HQ-RMs. The CV of the HQ-MSCs exhibited a pair of peaks at 0.15 V, which is a significant characteristic of the electrochemical multiple Faradaic redox reaction of the HQ-RMs during the charge/discharge cycles. The expected redox reactions of the HQ-RMs during the charge/discharge cycles are as follows (Figure 3b) [23,41,43,45]:

hydroquinone (HQ) → benzoquinone (BQ) 2H^+^ + 2e^−^ (charge process)

benzoquinone (BQ) 2H^+^ + 2e^−^ → hydroquinone (HQ) (discharge process)

Furthermore, the CV curves of the HQ-MSCs showed similar shapes with increasing scan rates from 10 to 100 mV s^−1^, indicating that the HQ-MSCs have good energy-storing kinetics and reversible capacitive behavior (Figure 3c). Especially, as indicated in Figure S3, at slow scan rates, all the possible ion adsorption and electrochemical reactions are maximized on the surface within the given sweeping window (the clear redox pairs are detected). However, at the fast scan rates, the relatively broad redox peaks can be observed as shown in Figure 3c and Figure S3, which are the normally recognized CV response of Faradaic redox materials. Furthermore, we carried out the CV tests to confirm the effects of the concentration of HQ in the MSCs. As shown in Figure S4, the HQ-MSCs with a HQ concentration of 0.135 M showed the low energy storing performance, which is 8.1 times lower than that of HQ-MSCs with the HQ concentration of 0.27 M. Furthermore, the enclosed CV areas of HQ-MSCs with an HQ concentration of 0.54 M (the maximum aqueous solubility) is also smaller than that of HQ-MSCs with an HQ concentration of 0.27 M. The excess amount of HQ-RMs in the electrolyte can decrease the overall electrochemical performance due to the low ionic conductivity and ion permeability through the gel electrolyte [44].

The galvanic charge/discharge graph (GCD) of the HQ-MSCs presented a longer discharge time than that of the b-MSCs (Figure 3d,e). The calculated area capacitance of the HQ-MSCs (2.58 mF cm^−2^) was approximately 2.7 times higher than that of the B-MSCs (0.95 mF cm^−2^) and other previously reported studies (summarized in Figure S5a). In addition, the volumetric capacitance of HQ-MSCs is 255 mF cm^−3^. The improved energy storage properties of HQ-MSCs were attributed to the additional Faradaic redox reactions of the HQ-RMs compared to those of the bare-gel electrolyte composites. In addition, as mentioned in the introduction, self-discharge in carbon-based micro-supercapacitors is another important issue that must be addressed to develop high-performance MSCs. To compare the self-discharging rate between HQ-MSCs and b-MSCs, we measured the voltage drop based on the rest time from the fully charged state of MSCs. As shown in Figure 3f, the HQ-MSCs exhibited a low self-discharge rate. The open circuit voltage drop rate of the HQ-MSCs was 37% after 2000s, which was lower than that of b-MSCs (50%) and other previously reported studies (summarized in Figure S5b). The charged ions formed by the electrochemical Faradaic redox reaction of the HQ-RMs were strongly adsorbed on the electrodes and had a low free diffusion rate into the bulk electrolyte under the polymer-gel electrolyte; therefore, the HQ-MSCs can exhibit low self-discharge rate behavior. Previous studies reported that a polymer–gel electrolyte with limited moisture exhibited a superiorly suppressed self-discharge rate compared to aqueous electrolytes because the limited moisture condition decreased the level of ion mobility from the surface of the electrode to the bulk electrolyte solution [46,47,48,49,50]. In addition, the charged species of the HQ-RMs formed during electrochemical capacitive behavior were adsorbed on the electrode surface, thereby suppressing the self-discharge process [49]. When the self-discharge rate of the MCSs was tested under the aqueous electrolyte solution with HQ, the open circuit voltage drop rate of the HQ-MSCs exhibited a rapid voltage drop within 200 s, as shown in Figure S6.

**Figure 4:**
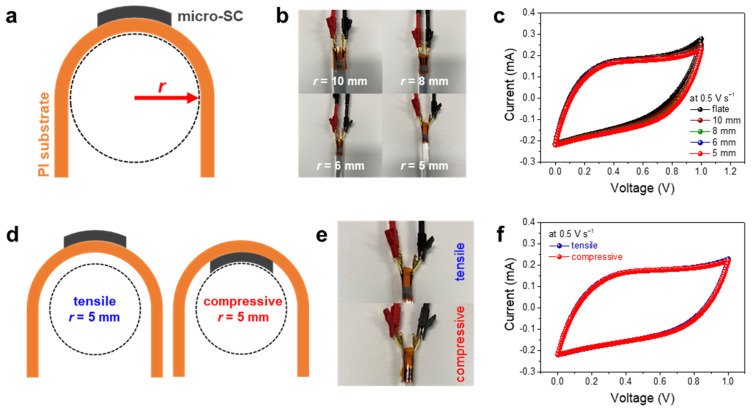
(**a**) Schematic illustration of bending states for HQ-MSCs. (**b**) Photographs and (**c**) CV curves of various bending states with bending radii of 5, 6, 8, and 10 mm for HQ-MSCs. (**d**) Schematic illustration and (**e**) photographs of HQ-MSCs under tensile and compressive strains. (**f**) CV curves of HQ-MSCs under tensile and compressive strains.

Furthermore, the mechanical flexibility and stability of the HQ-MSCs were estimated under different external strain levels (measured radius of curvature). The levels of external strains were normalized by the radius of curvature from 5 to 10 mm (Figure 4a,b). Figure 4c presents the CV curves of the HQ-MSCs when different levels of external strains were applied at a scan rate of 500 mV s^−1^. The CV curves did not show any significant changes during the strain tests, indicating its superior mechanical flexibility and stability against external strains. Furthermore, the HQ-MSCs exhibited a superior mechanical stability with a capacitance retention of 99.1% during the 1000 bending cycles (Figure S7). In addition, the mechanical stability of the HQ-MSCs against the tensile and compressive strains (at a radius of curvature of 5 mm) was analyzed (Figure 4d). Optical images of the HQ-MSCs under tensile and compressive strains are shown in Figure 4e. The CV curves at a scan rate of 500 mV s^−1^ under the tensile and compressive strains also have a similar shape to the CV curves without any significant curve distortion. After the diverse external strain tests, it can be clearly confirmed that the HQ-MSCs can be successfully applied to wearable devices owing to their high flexibility and performance stability against external forces.

**Figure 5:**
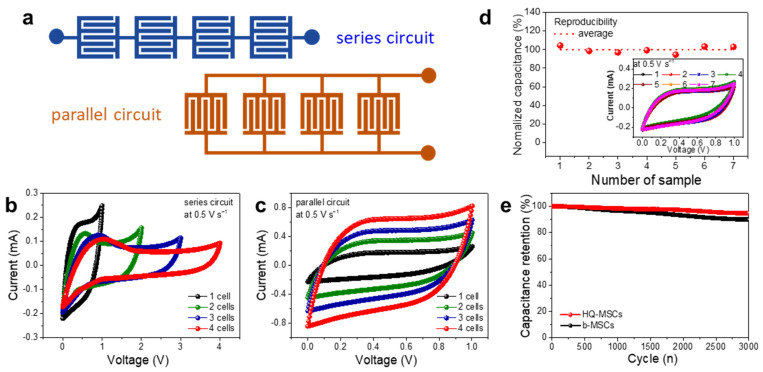
(**a**) Schematic illustration of series and parallel circuits for HQ-MSCs. CV curves of HQ-MSCs connected in (**b**) series and (**c**) parallel circuits. (**d**) Reproducibility and (**e**) cyclability tests of HQ-MSCs (the inset of (**d**) indicates CV curves obtained for seven HQ-MSCs devices).

To evaluate the circuit applicability of the HQ-MSCs, four different HQ-MSCs were assembled in series and parallel (Figure 5a). With the series or parallel connection, the operating cell voltage or capacitance is expected to increase proportionally according to the number of connected HQ-MSCs. Figure 5b shows the CV curves when the HQ-MSCs were connected in a series circuit. The voltage windows of 1 cell to 4 cells in series increased from 1 to 4 V, respectively. In addition, the CV currents increased proportionally based on the currents from 0.17 to 0.63 mA at 0.4 V when the HQ-MSCs were connected in parallel (Figure 5c). These CV results in the series and parallel circuits demonstrated great circuit operation of the HQ-MSCs when they were utilized as potential future energy-storing devices. Moreover, as shown in Figure 5d, HQ-MSCs show great reproducibility in energy-storing performance owing to their programmed fabrication process based on the laser scribing method of MSCs. The HQ-MSCs exhibited a promising capacitive retention behavior of 95% after 3000 cycles (Figure 5e).

## 4. Conclusions

In this study, we fabricated carbon-based micro-supercapacitors with an HQ-RM gel electrolyte using the laser scribing method for electrode patterning and drop-coating for MSC assembly. The carbon-based interdigitated electrodes formed using the laser scribing method show graphitic carbon crystalline features with high electrical conductivity and a porous structure. In terms of electrochemical features, HQ-MSCs have a high volumetric capacitance of 255 mF cm^−3^ at a current of 1 µA, which is 2.7 times higher than that of the b-MSCs, as well as a low self-discharge rate with an open circuit potential drop of 37% after 2000 s. The corresponding results are highly relevant for performing additional Faradaic redox reactions of the HQ-RMs and synergistically improve the overall energy storage performance. Moreover, the HQ-RMs in the gel electrolyte decrease the self-charging rate by providing a strong binding attachment of electrolyte ions on the surface of the electrodes. Furthermore, the HQ-MSCs displayed remarkably excellent mechanical features under various external mechanical stresses. Additionally, the HQ-MSCs exhibited a high reproducibility and a long-term cyclability with a high cycling capacitance retention of 95% after 3000 cycles. Therefore, the introduction of HQ redox mediators in micro-supercapacitor systems is a promising gel electrolyte additive for flexible high-performance energy storage applications.

## Supplementary Materials

The following are available online at https://www.mdpi.com/article/10.3390/nano11113027/s1, Figure S1: Cross-section SEM images of the fabricated MSCs, Figure S2: Photograph images of the measured two-probe electrical resistance: (left) the GCEs region and (right) the pure PI film for the patterned MSCs, Figure S3: CV curves of HQ-MSCs with different scan rates from 0.1 V s^−1^ and to 1.0 V s^−1^, Figure S4: CV curves of HQ-MSCs with different HQ concentrations of (a) 0.27 M and (b) 0.54 M at a scan rate of 1.0 V s^−1^, respectively, Figure S5: Comparison of the areal capacitance and self-discharge rate of the fabricated MSCs compared to other previously reported literatures, Figure S6: Comparison of self-discharging test of MSCs under gel electrolyte and aqueous electrolyte with HQ after fully charged state, Figure S7. Capacitance retention of HQ-MSCs for 1000 bending cycles (inset indicates the CV curves of HQ-MSCs).
