# Supplementary material for: A Redox-Mediator-Integrated Flexible Micro-Supercapacitor with Improved Energy Storage Capability and Suppressed Self-Discharge Rate
Source: Nanomaterials (Basel). 2021 Nov 11;11(11):3027. doi: 10.3390/nano11113027 (PMC8624181; doi:10.3390/nano11113027)
Supplement: Supplementary file 1 [file nanomaterials-11-03027-s001.zip › nanomaterials-1440122-supplementary.pdf]

Supplementary Materials

A Redox-Mediator-Integrated Flexible Micro-Supercapacitor with Improved Energy Storage Capability and Suppressed Self-Discharge Rate

Sung Min Wi [1,†], Jihong Kim [1,†], Suok Lee [1,†], Yu-Rim Choi [1], Sung Hoon Kim [1], Jong Bae Park [2], Younghyun Cho [1], Wook Ahn [1], A-Rang Jang [3], John Hong [4,*] and Young-Woo Lee [1,*]

[1] Department of Energy Systems Engineering, Soonchunhyang University, Asan-si 31538, Korea; dnlals77@naver.com (S.M.W.); colorg11@naver.com (J.K.); solee0117@gmail.com (S.L.); uj1008@sch.ac.kr (Y.-R.C.); nyk4123@naver.com (S.H.K.); yhcho@sch.ac.kr (Y.C.); wahn21@sch.ac.kr (W.A.)

[2] Jeonju Centre, Korea Basic Science Institute, Jeonju 54907, Korea; jbpjb@kbsi.re.kr

[3] Department of Electrical Engineering, Semyung University, Jecheon-si 27136, Korea; arjang@semyung.ac.kr

[4] School of Materials Science and Engineering, Kookmin University, Seoul 02707, Korea

* Correspondence: johnhong@kookmin.ac.kr (J.H.); ywlee@sch.ac.kr (Y.-W.L.); Tel.: +82-2-910-4665 (J.H.); +82-41-530-4988 (Y.-W.L.)

† These authors contributed equally to this work.

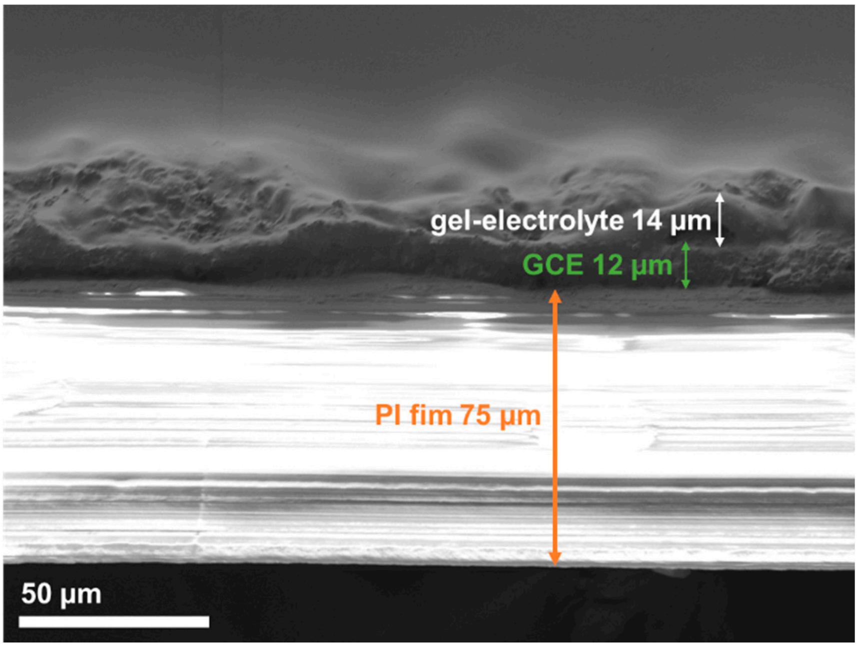

Figure S1. Cross-section SEM images of the fabricated MSCs.

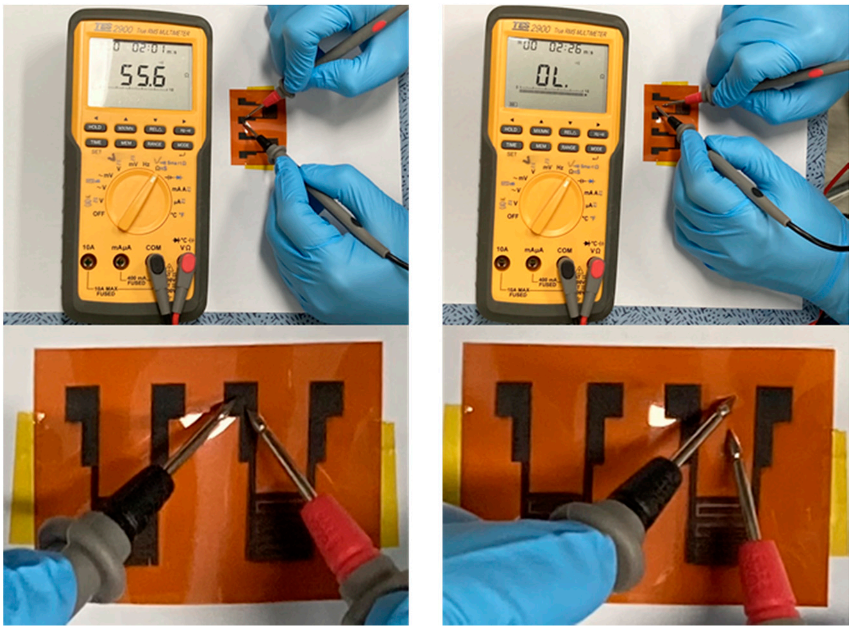

Figure S2. Photograph images of the measured two-probe electrical resistance: (left) the GCEs region and (right) the pure PI film for the patterned MSCs.

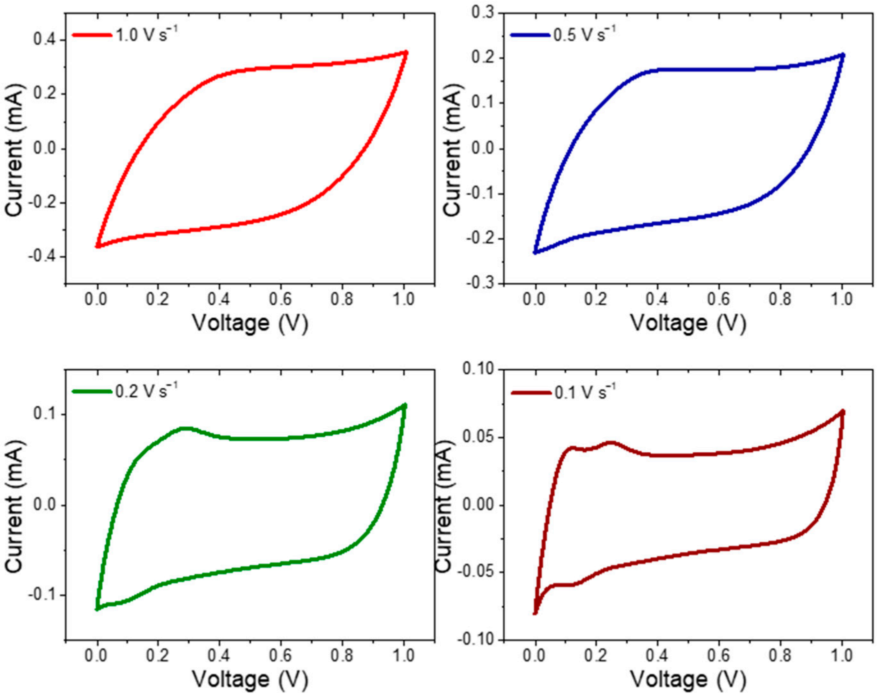

Figure S3. CV curves of HQ-MSCs with different scan rates from 0.1 V s^{-1} and to 1.0 V s^{-1}.

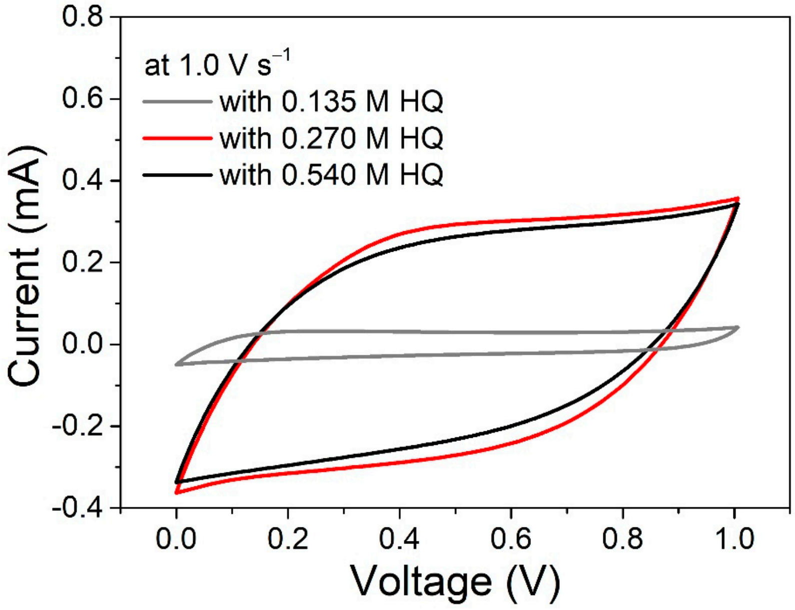

Figure S4. CV curves of HQ-MSCs with different HQ concentrations of 0.135 M, 0.270 M, and 0.540 M at a scan rate of 1.0 V s^{-1}, respectively.

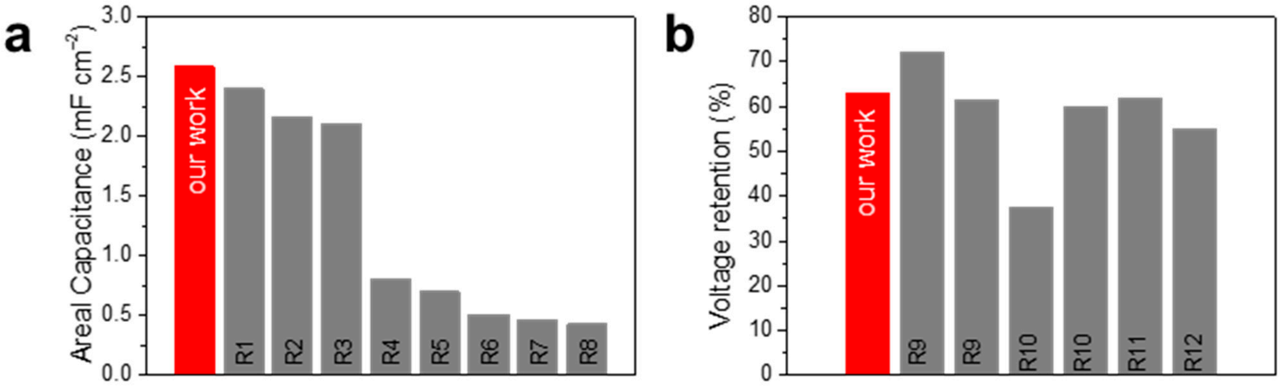

Figure S5. Comparison of the areal capacitance and self-discharge rate of the fabricated MSCs compared to other previously reported literatures.

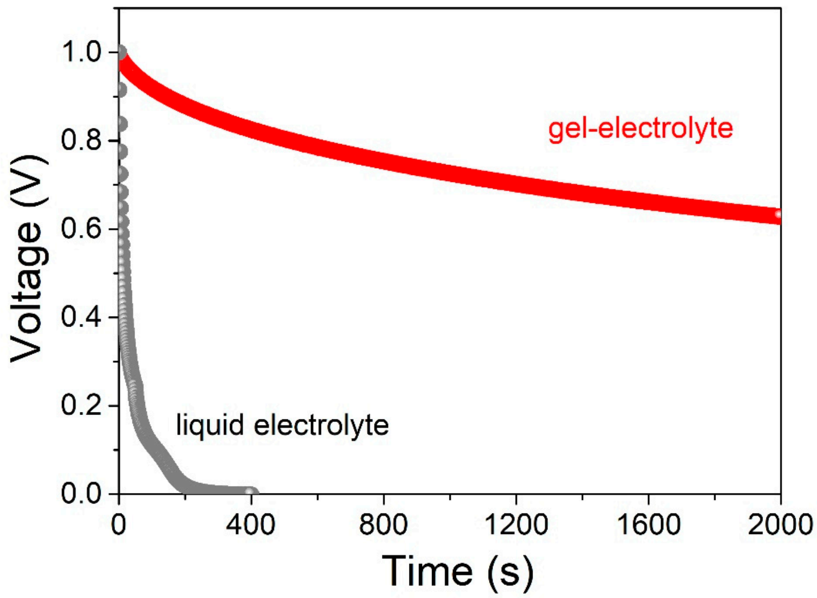

Figure S6. Comparison of self-discharging test of MSCs under gel electrolyte and aqueous electrolyte with HQ after fully charged state.

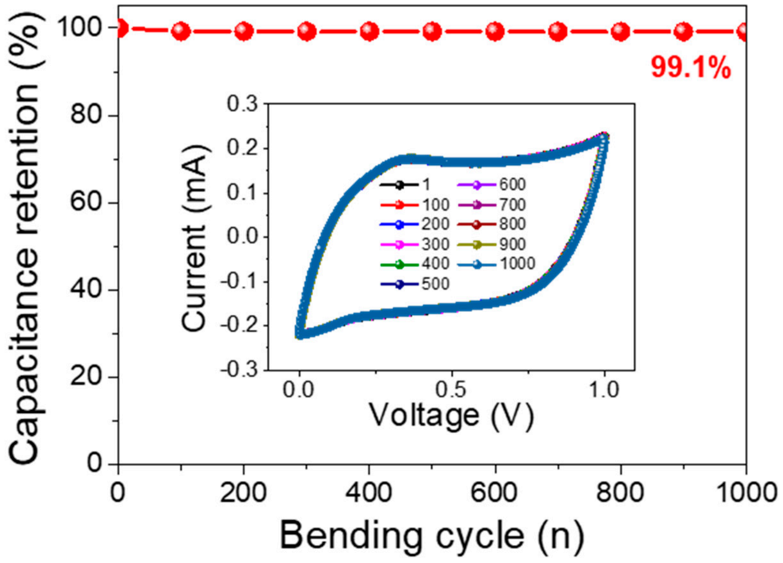

Figure S7. Capacitance retention of HQ-MSCs for 1000 bending cycles (inset indicates the CV curves of HQ-MSCs).

References

R1. Couly, C.; Alhabeb, M.; Van Aken, K. L.; Kurra, N.; Gomes, L.; Navarro-Suarez, A. M.; Anasori, B.; Alshareef, H. N.; Gogotsi, Y. Asymmetric flexible MXene-reduced graphene oxide micro-supercapacitor. *Adv. Electron. Mater.* **2018,** *4*, 1700339. https://doi.org/10.1002/aelm.201700339

R2. Lin, J.; Zhang, C.; Yan, Z.; Zhu, Y.; Peng, Z,; Hauge, R. H.; Natelson, D.; Tour, J. M. 3-dimensional graphene carbon nanotube carpet-based microsupercapacitors with high electrochemical performance. *Nano Lett.* **2013,** *13*, 72-78. https://doi.org/10.1021/nl3034976

R3. Pech, D.; Brunet, M.; Taberna, P.-L.; Simon, P.; Fabre, N.; Mesnilgrente, F.; Conedera, V.; Durou, H. Elaboration of a microstructured inkjet-printed carbon electrochemical capacitor. *J. Power Sources* **2010,** *195,* 1266-1269. http://doi.org/10.1016/j.jpowsour.2009.08.085

R4. In, J. B.; Hsia, B.; Yoo, J.-H.; Hyun, S.; Carraro, C.; Maboudian, R.; Grigoropoulos, C. P. Facile fabrication of flexible all solid-state micro-supercapacitor by direct laser writing of porous carbon in polyimide. *Carbon* **2015,** *83,* 144-151. http://doi.org/10.1016/j.carbon.2014.11.017

R5. Li, J.; Delekta, S. S.; Zhang, P.; Yang, S.; Lohe, M. R.; Zhuang, X.; Feng, X.; Ostling, M. Scalable fabrication and integration of graphene microsupercapacitors through full inkjet printing. *ACS Nano* **2017,** *11,* 8249-8256. http://doi.org/10.1021/acsnano.7b03354

R6. Gao, W.; Singh, N.; Song, L.; Reddy, A. L. M.; Ci, L.; Vajtai, R.; Zhang, Q.; Wei, B.; Ajayan, P. M. Direct laser writing of micro-supercapacitors on hydrated graphite oxide films. *Nat. Nanotechnol.* **2011,** *6,* 496-500. https://doi.org/10.1038/nnano.2011.110

R7. Liu, W.-W.; Feng, Y.-Q.; Yan, X.-B.; Chen, J.-T.; Xue, Q.-J. Superior Micro-Supercapacitors Based on Graphene Quantum Dots. *Adv. Funct. Mater.* **2013,** *23,* 4111-4122. https://doi.org/10.1002/adfm.201203771

R8. Jiang, Y.Q.; Zhou, Q.; Lin, L. Planar MEMS supercapacitor using carbon nanotube forests. *2009 IEEE 22nd International Conference on Micro Electro Mechanical Systems.* **2009,** 587-590. http://doi.org/10.1109/MEMSYS.2009.4805450

R9. Tevi, T.; Yaghoubi, H.; Wang, J.; Takshi, A. Application of poly (p-phenylene oxide) as blocking layer to reduceself-discharge in supercapacitors. *J. Power Sources* **2013,** *241,* 589-596. https://doi.org/10.1016/j.jpowsour.2013.04.150

R10. Chen, L.; Bai, H.; Huang, Z.; Li, L. Mechanism investigation and suppression of self-discharge in active electrolyte enhanced supercapacitors. *Energy Environ. Sci.* **2014,** *7,* 1750-1759. https://doi.org/10.1039/C4EE00002A

R11. Fan, L.-Q.; Tu, Q.-M.; Geng, C.-L.; Huang, J.-L.; Gu, Y.; Lin, J.-M.; Huang, Y.-F.; Wu, J.-H. High energy density and low self-discharge of a quasi-solid-state supercapacitor with carbon nanotubes incorporated redox-active ionic liquid-based gel polymer electrolyte. *Electrochim. Acta* **2020,** *331,* 135425. https://doi.org/10.1016/j.electacta.2019.135425

R12. Liu, J.-H.; Xu, X.-Y.; Liu, C.; Chen, D.-Z. Thermal effect on the pseudocapacitive behavior of high-performance flexible supercapacitors based on polypyrrole-decorated carbon cloth electrodes. *New J. Chem.* **2021,** *45,* 12435-12447. https://doi.org/10.1039/D1NJ01513C
